# Enhancing Yeast Transformation: Achieving up to a Tenfold Increase Through a Single Adjustment in the Lithium Acetate–Polyethylene Glycol Method

**DOI:** 10.1002/yea.3999

**Published:** 2025-03-15

**Authors:** Mathilde Kadouch, Pierre Gaspin, Christelle Marchal, Sabine Castano, Christophe Cullin

**Affiliations:** ^1^ Univ. Bordeaux, CNRS, Bordeaux INP, CBMN, UMR 5248 Pessac France

**Keywords:** Lithium–PEG, sorbitol, yeast transformation

## Abstract

The Lithium–PEG method for transforming yeast cells is a standard procedure used in most yeast laboratories. After several optimizations, this method can yield up to 10^6^ transformants per µg of plasmid. Some applications, such as library screening or complex transformations, necessitate maximizing transformation yield. Here, we demonstrate that the addition of a sorbitol solution serves as an osmo‐protectant during and after heat shock, resulting in up to a tenfold increase in transformation efficiency. This optimization requires only one additional pipetting step compared to the original protocol, making it practical for routine use.

## Summary

- Adding a sorbitol solution can enhance transformation efficiency by up to tenfold.
- Sorbitol solution enhances cell viability by reducing osmotic stress.

## Graphical abstract

In red are the proposed improvements to the classical yeast transformation protocol.

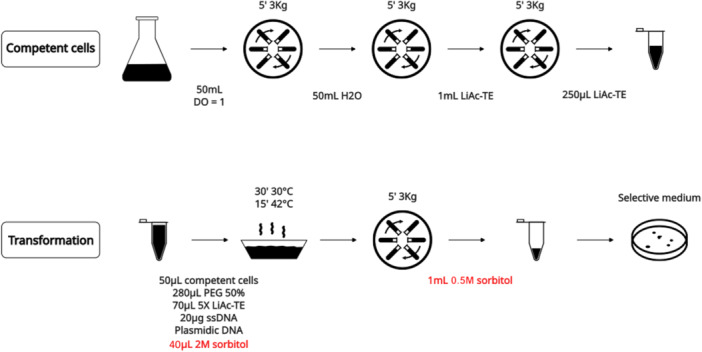

## 1 Introduction

Yeast transformation is a fundamental experiment for any scientist working with *Saccharomyces cerevisiae*. Most, if not all, yeast laboratories routinely use the PEG–LiAc method. This choice is driven by its simplicity (requiring only a centrifuge and a thermostatic bath), cost‐effectiveness (involving only inexpensive chemical reagents), ecological benefits (no plastic waste or transport of a “kit‐box”), and versatility (compatible with DNA in high‐salt buffers and mixed with proteins, etc.).

In contrast to the initial protocol based on protoplast transformation (Beggs 1978), the PEG–LiAc method (Ito et al. 1983) is a rapid, robust, and straightforward procedure that consistently yields transformed yeast cells. The original protocol has been optimized to achieve yields of up to 10^5^ transformants per µg of plasmid (Schiestl and Gietz 1989). The PEG–LiAc method provides comparable, if not superior, transformation efficiency compared to the spheroplast method, establishing it as the preferred choice for yeast transformations.

Several parameters—including plasmid and carrier DNA concentration, cell density, and heat‐shock duration—can be adjusted to enhance the efficacy of this protocol (Gietz et al. 1995). However, in all these protocols, the pelleted cells are resuspended in water (or another hypotonic solution such as YPD) prior to plating onto selective media. Under physiological conditions, this treatment does not compromise cell viability. Nevertheless, since lithium acetate, ssDNA, and PEG are known to increase yeast cell permeability (Kawai et al. 2010; Chen et al. 2008) and also change cell wall morphology (Pham et al. 2011), it may also impact cell viability in hypotonic environments. Here, we provide evidence that this is indeed the case. We investigated both viability and transformation efficiency under osmo‐protective conditions and propose a modified protocol. This modified protocol, which requires only one additional pipetting step and preparation of a sorbitol solution, can increase the number of transformants by up to tenfold using BY or W303‐1b strains.

## 2 Results

### 2.1 Competent Yeast Cells Are Sensitive to Osmotic Stress After Heat‐Shock

In the original protocol by Schiestl and Gietz (1989), yeast cells are centrifuged after incubation at 42°C, washed in TE buffer, and plated on agarose plates. Their optimized method, which achieves yields of up to 10^6^ transformants per µg of plasmid DNA (Gietz et al. 1992), includes the same final steps. Subsequent iterations of this efficient protocol proposed by Gietz and Schiestl (2007a, 2007b) also incorporate the washing step, although sterile water is used instead of TE for plating the yeast cells.

**Figure 1:**
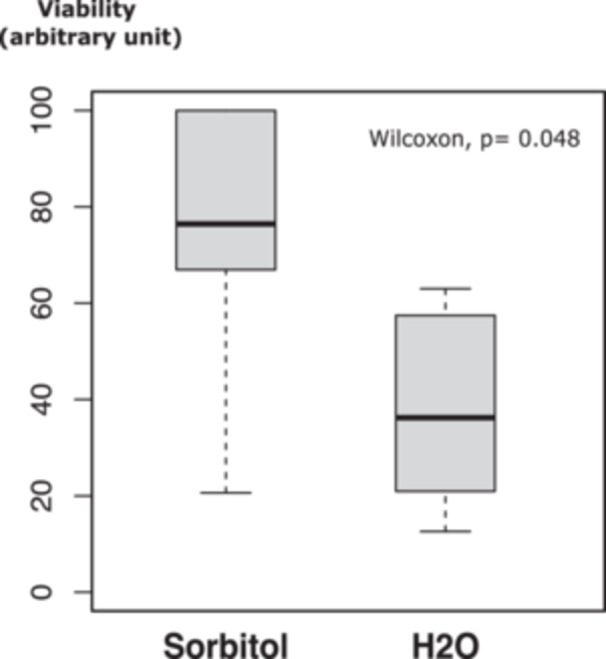
Influence of sorbitol on viability. Competent heat‐shocked yeast cells were divided into two equal fractions and resuspended either in 2 M sorbitol or in sterile water. Cellular viability was determined by plating onto complete medium after serial dilution in 2 M sorbitol or H_2_O. For each of the two experiments comprising six replicates, the higher value is set to 100 and the other CFU are expressed as a percentage of this maximum.

In all these protocols, yeast cells are washed in a hypotonic solution. To assess the resilience of the permeabilized yeast cells under these conditions, we examined their viability in water versus 2 M sorbitol. In one experiment, six aliquots (50 µL each) of BY4742∆TRP1 yeast cells, resuspended in LiAc–TE, were incubated for 30 min at 30°C in the presence of ssDNA and PEG, followed by a heat shock for 15 min at 42°C. After centrifugation and removal of the PEG/LiAc–TE/ssDNA mixture, the pelleted cells were resuspended in either 1 mL of sterile water or 1 mL of 2 M sorbitol. Following serial dilution (in water or sorbitol), two aliquots (100 µL each) of cells were plated on YPD medium. The number of colonies was counted after 3 days of incubation at 30°C. This experiment was repeated twice with two independent yeast cultures, and Table 1 presents the average values. The results (Figure 1) indicate a significant loss of viability when treated yeast cells are resuspended in water, as evidenced by the ~50% reduction in colony formation compared to yeast cells resuspended in 2 M sorbitol (Wilcoxon rank sum test, *W* = 31, *p* value = 0.048).

**Table 1:** Influence of sorbitol on viability.

	Experiment 1: cells resuspended in		Experiment 2: cells resuspended in	
	Sorbitol	H_2_O	Sorbitol	H_2_O
Viability (cfu on YPD × 10^5^)	223 (72)	102 (33)	127 (100)	80 (63)
	310.5 (100)	123 (37)	85 (67)	16 (13)
	64 (21)	65 (21)	103 (81)	73 (57)
Mean	199 (64)	97 (31)	105 (83)	56 (44)

*Note:* After permeabilization and heat shock, yeast cells were divided into two equal fractions, centrifuged, and resuspended in either 2 M sorbitol or H_2_O. Cell viability was assessed by plating onto complete medium after serial dilutions in either 2 M sorbitol or H_2_O. For each experiment, the higher value was set to 100, and the colony‐forming units (CFUs) of the other fraction are expressed as a percentage of this maximum value, resulting in the numbers indicated in brackets (rounded to the nearest integer).

### 2.2 Yeast Cells Are Sensitive to Osmotic Stress When Acquiring Their Competence

Next, we investigated the osmotic sensitivity of yeast cells during incubation in the presence of PEG. Two solutions of 50% PEG were utilized: the first (S) corresponded to the standard protocol (50% PEG in water), while the second (S*) was prepared in sorbitol, achieving a final concentration of 1.2 M. Additionally, we supplemented the standard mix (50% PEG in water) with either 90 µL of water (S + H) or 2 M sorbitol (S + S).

Yeast cells were incubated in these various mixtures for 30 min at 30°C in the presence of ssDNA and plasmid DNA (80 ng) before undergoing a heat shock for 15 min at 42°C. In all cases, the transformed yeast cells were resuspended in 2 M sorbitol. We assessed both cellular viability (by plating on complete medium) and transformation efficiency (by plating onto selective medium).

**Figure 2:**
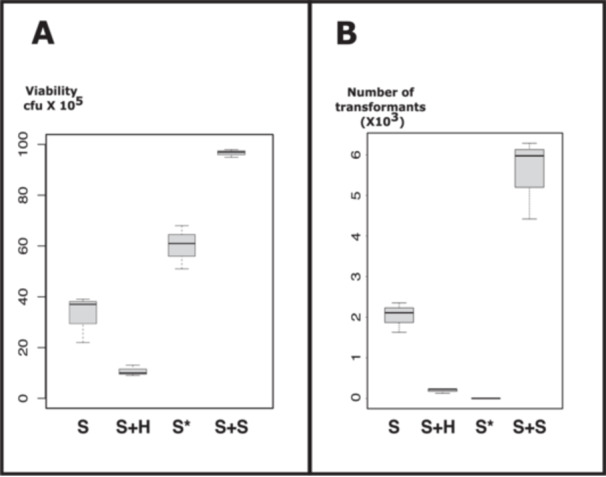
Influence of sorbitol on transformation rates. (A) Competent cells were subjected to various PEG solution mixtures before heat shock. They were resuspended in 2 M sorbitol, and cellular viability was determined by plating onto complete medium after serial dilution in 2 M sorbitol. (B) Competent cells were subjected to different PEG solution mixtures prior to heat shock. They were resuspended in 2 M sorbitol, and transformation rates were assessed by plating onto selective medium after serial dilution in 2 M sorbitol. S: Standard (356 µL PEG 40%, AcLi 0.1 M, TE 1X, 20 µg ssDNA, 100 ng plasmid). S + H: Standard + 90 µL H_2_O. S*: Modified standard mix with PEG in sorbitol instead of water (final concentration of 1.2 M sorbitol). S + S: Standard + 90 µL 2 M sorbitol.

Table 2 presents the values plotted in Figure 2. Notably, the addition of water (i.e., reducing osmotic pressure) resulted in decreased cellular viability (S vs. S + H, panel A). Conversely, the addition of sorbitol enhanced viability (S + S vs. S, panel A). This trend was also observed, albeit to a lesser extent, with the modified mix (50% PEG, 1.2 M sorbitol) (S* vs. S, panel A).

**Table 2:** Influence of sorbitol on viability and transformation efficiency.

	S	S + H	S*	S + S
Viability (cfu on YPD × 10^5^)	39	9	61	98
	22	13	51	97
	37	10	68	95
Mean	33	11	60	97
Number of transformants (cfu on selective medium)	2350	225	0	4420
	1630	230	0	6285
	2110	125	0	5975
Mean	2030	193	0	5560

*Note:* Competent yeast cells were incubated with four different mixes and then heat‐shocked. Transformed cells were resuspended in 2 M sorbitol. Viability of yeast cells during permeabilization was determined by plating onto complete medium. The transformation efficiency was determined by plating on selective medium. S: standard mix (356 µL PEG 40%, LiAc 0.1 M, TE 1X, 30 µg ssDNA, 100 ng plasmid); S + H: standard + 90 µL H_2_O; S*: 356 µL modified standard mix with PEG in sorbitol instead (final concentration of 1.2 M sorbitol); S + S: standard + 90 µL 2 M sorbitol.

Transformation efficiency exhibited a similar pattern, except for one condition: S* (panel B). Surprisingly, at high concentrations of sorbitol and PEG, we were unable to get any transformants. This complete inhibition of transformation is reproducible. However, the addition of sorbitol to the standard mix significantly improved transformation efficiency (S + S vs. S, panel B).

### 2.3 Optimization of Sorbitol Concentration

**Figure 3:**
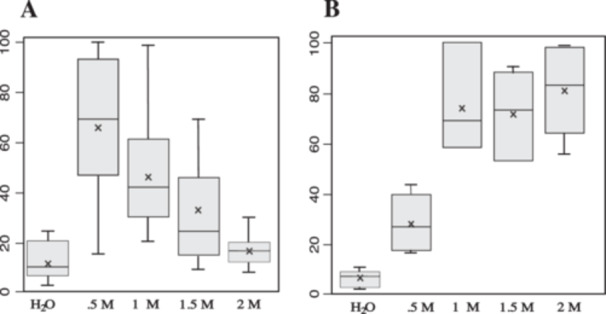
Optimization of sorbitol concentration. (A) Cell viability. After heat‐shock, yeast cells were resuspended in H_2_O or sorbitol at different concentrations. Cell viability was assessed by plating onto complete medium after serial dilutions in isotonic buffer. For each experiment, the higher value was set to 100, and the colony‐forming units (CFUs) of the other fraction are expressed as a percentage of this maximum value. (B) Transformation rate. In total, 90 µL of H_2_O or sorbitol at different concentrations were added to the transformation mix of PEG–LiAc–TE–ssDNA and plasmid. After heat‐shock, yeast cells were resuspended in 1 mL of 0.5 M sorbitol. Transformation efficiency was determined by plating 50 µL on selective medium. For each experiment, the higher value was set to 100, and the colony‐forming units (CFUs) of the other fraction are expressed as a percentage of this maximum value.

Since sorbitol enhances cell viability, we subsequently measured this effect at various concentrations. In a first set of experiments, we compared the sensitivity of competent cells to solutions ranging from 0 to 2 M sorbitol. Six aliquots (50 µL each) of BY4742∆TRP1 yeast cells, resuspended in LiAc–TE, were incubated for 30 min at 30°C in the presence of ssDNA, LiAc–TE, and PEG, followed by a heat shock for 15 min at 42°C. After centrifugation and removal of the PEG/LiAc–TE/ssDNA mix, the pelleted cells were resuspended in either 1 mL of sterile water or sorbitol at different concentrations (0.5, 1, 1.5, and 2 M). Following serial dilution (in water or sorbitol), three or four aliquots (50 µL each) of cells were plated on YPD medium. The number of colonies was counted after 3 days of incubation at 30°C. The results presented in Table 3 derive from three independent assays. The corresponding box‐plot (Figure 3A) illustrates the protective role of sorbitol, with the most effective concentration being 0.5 M.

**Table 3:** Optimization of sorbitol concentration for cell recovery.

		Yeast cells resuspended with …				
		H_2_O	0.5 M	1 M	1.5 M	2 M
Viability (cfu on YPD × 10^5^)	Experiment 1	15 (4)	406 (100)	125 (31)	58 (14)	79 (20)
		37 (9)	135 (33)	234 (58)	135 (33)	122 (30)
		40 (10)	305 (75)	135 (33)	61 (15)	57 (14)
	Experiment 2	115 (25)	225 (48)	144 (31)	157 (34)	59 (13)
		98 (21)	279 (60)	360 (77)	140 (30)	40 (9)
		14 (3)	338 (73)	460 (99)	320 (69)	52 (11)
		59 (13)	465 (100)	207 (45)	320 (69)	116 (25)
	Experiment 3	12 (11)	52 (47)	23 (21)	19 (17)	20 (18)
		23 (21)	18 (16)	30 (27)	17 (15)	18 (16)
			110 (100)	58 (53)	11 (10)	20 (18)

*Note:* After permeabilization and heat shock, yeast cells were divided into five equal fractions, centrifuged, and resuspended in H_2_O or sorbitol at different concentrations. Cell viability was assessed by plating onto complete medium after serial dilutions in isotonic buffer. For each experiment, the higher value was set to 100, and the colony‐forming units (CFUs) of the other fraction are expressed as a percentage of this maximum value, resulting in the numbers indicated in brackets (rounded to the nearest integer).

Next, we optimized the concentration of the sorbitol added in the transformation mix. Competent yeast cells (50 µL) were incubated with the standard mix (356 µL PEG 40%, LiAc– TE, 25 µg ssDNA, 80 ng plasmid) + 90 µL of H_2_O or sorbitol at different concentrations. After heat‐shock, yeast cells were resuspended in 1 mL of 0.5 M sorbitol. The transformation efficiency was determined by plating several aliquots of 50 µL on selective medium. The results presented in Table 4 derive from two independent experiments with two different cultures. The corresponding box‐plot (Figure 3B) demonstrates a protective role for sorbitol. This protective effect remains roughly the same at concentration ranging from 1 to 2 M.

**Table 4:** Optimization of sorbitol concentration added in the transformation mix.

		Yeast cells incubated at 42°C with 90 µL of				
		H_2_O	0.5 M	1 M	1.5 M	2 M
Number of clones in selective medium (50 µL)	Experiment 1	2 (3)	11 (17)	32 (51)	57 (90)	42 (67)
		5 (8)	16 (25)	44 (70)	53 (84)	35 (56)
		1 (2)	24 (38)	63 (100)	55 (87)	49 (78)
	Experiment 2	11 (7)	27 (17)	162 (100)	101 (62)	143 (88)
		11 (7)	44 (27)	110 (68)	85 (52)	158 (98)
		17 (10)	70 (43)	97 (60)	85 (52)	160 (99)

*Note:* Competent yeast cells (50 µL) were incubated with the standard mix (356 µL PEG 40%, LiAc 0.1 M, TE 1X, 30 µg ssDNA, 80 ng plasmid) + 90 µL of H_2_O or sorbitol at different concentrations. After heat‐shock, yeast cells were resuspended in 1 mL of 0.5 M sorbitol. The transformation efficiency was determined by plating 50 µL on selective medium. For each experiment, the higher value was set to 100, and the colony‐forming units (CFUs) of the other fraction are expressed as a percentage of this maximum value, resulting in the numbers indicated in brackets (rounded to the nearest integer).

We combined our two observations—protection by sorbitol during incubation and during cell recovery—and compared this optimized protocol with the standard protocol.

### 2.4 Two Slight Modifications Improve Yeast Transformation Rate

The modified protocol was assessed with two yeast strains: W303‐1b and BY4742ΔTRP1. For each assay, 50 mL of cell culture (OD_650nm_≈1) was collected, washed, and resuspended in 250 µL of a buffer containing lithium acetate (LiAc, 0.1 M), Tris‐HCl (50 mM, pH 7.4), and EDTA (1 mM). In the standard condition, 50 µL of competent cells were mixed with 350 µL of a solution, consisting of 280 µL of 50% polyethylene glycol (PEG) and 70 µL of 5X LiAc–TE, along with 5 µL of DNA carrier (25 µg) and 1 µL of plasmid DNA (80 ng). After heat shock, the yeast cells were suspended either in 1 mL of water (H) or in 1 mL of 0.5 M sorbitol (S).

In parallel, 50 µL of competent cells were mixed with 350 µL of the standard solution in which 90 µL of 2 M sorbitol was added. After heat shock, the yeast cells were suspended in 1 mL of 0.5 M sorbitol (SS).

A volume of 100 µL from each suspension was then spread onto selective medium, with several plates prepared for each experimental condition. The modified protocol incorporating sorbitol (SS) demonstrated a significant enhancement in transformation efficiency compared to the standard protocol (H) across a dozen independent experiments. This improvement was consistent regardless of the yeast strain used (data not shown). However, the level of improvement was highly variable ranging from 2 to 10. This lack of reproducibility coincides each time with the use of new PEG or sorbitol solutions. We checked the impact of the water, the sterilization process (filtration vs. sterilization) and the origin of the chemical products, but were unable to find any explanation for this discrepancy. In all cases, the greatest effect observed was due to the recovery of cells in a 0.5 sorbitol solution (S vs. H vs. SS). We therefore decided to analyze the results using the batch of compounds that led to the least favorable outcome. We also adjusted the volume of 2 M sorbitol added to the standard mix (40 µL instead of 90 µL) to maintain a protective effect while retaining a high PEG concentration. Table 5 shows the results obtained with three independent cultures of BY4742ΔTRP1 and three independent cultures of W303‐1b.

**Table 5:** Efficacy of the modified protocol.

	BY4742 Δ*TRP1*			W303 1‐B		
	H	S	SS	H	S	SS
Number of transformants (cfu 10%)	366 (53)	313 (45)	440 (64)	8 (4)	22 (11)	128 (66)
	393 (57)	520 (75)	692 (100)	10 (5)	34 (18)	136 (70)
	337 (49)	422 (61)	541 (78)	12 (6)	26 (13)	194 (100)
	161 (31)	436 (83)	249 (47)	42 (11)	84 (21)	266 (67)
	67 (13)	374 (71)	344 (66)	24 (6)	54 (14)	400 (100)
	48 (9)	525 (100)	319 (61)	36 (9)	56 (14)	260 (65)
	246 (42)	453 (78)	435 (75)	50 (17)	266 (90)	290 (98)
	283 (49)	581 (100)	372 (64)	43 (15)	285 (96)	296 (100)
	240 (41)	549 (94)	401 (69)	56 (19)	262 (89)	257 (87)
	260 (45)	567 (98)		48 (16)	243 (82)	250 (84)
Mean	39	81	69	11	45	84

*Note:* Competent yeast cells (W303‐1b or BY4742ΔTRP1) were transformed using the standard protocol and plated in triplicate or quadruplicate after resuspension in H_2_O (H) or 0.5 M sorbitol (S). Alternatively, 40 µL of 2 M sorbitol was added in the transformation mix and cells were plated after resuspension in 0.5 M sorbitol (SS). Transformation efficiency, expressed as a percentage of the maximum colony‐forming units (CFUs) per experiment (rounded to the nearest integer, values in brackets), is shown for six independent experiments using three independent cultures of each strain.

**Figure 4:**
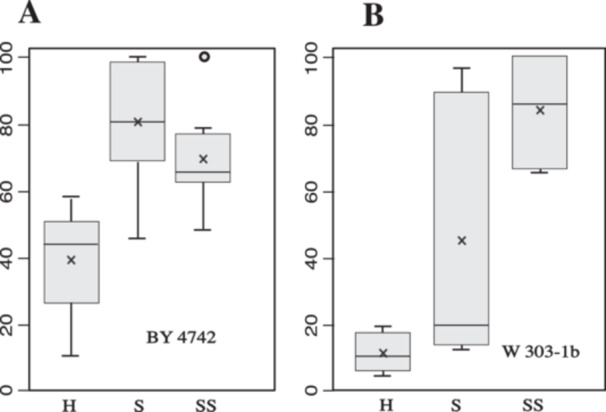
Efficacity comparison of standard versus modified protocol. For each of the six experiments, 50 mL of BY∆TRP1 (A) or W303‐1b (B) yeast culture at OD = 1 was rendered competent. Cells transformed with the classical protocol were suspended either in H_2_O (H) or 0.5 M sorbitol (S). Cells transformed with the modified protocol (by adding 40 µL of 2 M sorbitol in the transformation mix) were resuspended in 0.5 M sorbitol. For each experiment, the number of colonies is normalized to the maximum CFU observed and expressed as a percentage of this maximum rounded at the nearest integer.

The corresponding box‐plots (Figure 4) highlight the capacity for the sorbitol solution to increase transformation efficiency. Interestingly, the genetic background has a strong impact on the results. With strain BY4742ΔTRP1, the sorbitol added in the transformation mix has no significant effect (panel A SS vs. S) while the use of sorbitol for cell suspension increases by a factor of around two the transformation efficiency (H vs. S or SS). In contrast, W303‐1b is much more sensitive to sorbitol (panel B) and the optimized protocol increases transformation efficiency by a factor of around 8 (SS vs. H). Interestingly, adding sorbitol in the transformation mix seems to decrease the variability of the results (SS vs. S).

## 3 Discussion

We demonstrate that the classical LiAc treatment of yeast cells leads to a loss of viability in hypotonic solutions. This parameter can be adjusted by using sorbitol solutions during heat shock and during the recovery of the cell pellet prior to plating.

Surprisingly, we also show that when sorbitol is used to prepare the PEG solution (i.e., achieving the same final concentration of sorbitol without diluting the PEG), cell viability increases, while transformation is inhibited. The PEG–sorbitol solution is highly viscous, and this increase in viscosity might be partially responsible for the loss of competency. PEG alone also increases the osmotic pressure (Money 1989), and when combined with sorbitol, may lead to hyperosmotic conditions that can be detrimental to DNA uptake. When a small amount of sorbitol (40 µL of 2 M sorbitol solution) is added to the standard transformation mix, both cellular viability and transformation efficiency increase. Taken together, the osmotic protection provided by sorbitol during and after heat shock allows for a significant improvement (up to tenfold) in yeast transformation yield.

This optimized protocol requires only one additional pipetting step, making it easily implementable in yeast laboratories without significantly increasing the time spent on this common experiment. This increase in transformation yield can be of great interest, particularly when one wants to screen a library or to perform more complex transformation experiments.

## 4 Materials and Methods

### 4.1 Yeast Strains, Plasmid, and Growth Media

The two yeast strains used are W303‐1b {MATα leu2‐3,112 trp1‐1 can1‐100 ura3‐1 ade2‐1 his3‐11,15} and BY4742∆TRP1 {MATa his3Δ1 leu2Δ0 met15Δ0 ura3Δ0 YDR007W::kanMX}, obtained from the commercial EUROSCARF knockout library.

Transformations were performed using pAG424‐ccdB (Addgene), in which the ORF of the URE2 gene replaces the ccdb cassette, as described by Alberti et al. (2007).

Yeast strains were routinely grown in YPD (1% yeast extract, 2% peptone, and 2% glucose in distilled water), and transformed yeast was selected on SC‐TRP (CSM‐Trp (Formedium) 740 mg/L, YNB (DIFCO) 6.7 g/L, Glucose 2%, Agar 2%). With the exception of sorbitol (Formedium), all chemical products were sourced from Sigma.

### 4.2 Transformation

In total, 50 mL of YPD were inoculated with 1 mL of an overnight saturated culture (in YPD) and incubated at 30°C until the culture reached an OD_650nm_ of 1. The culture was centrifuged (5′ at 3000 rpm) and washed with 50 mL of sterile water. After centrifugation (5′, 3000 rpm), yeast cells were resuspended in 1 mL of LiAc–TE solution (0.1 M lithium acetate, 50 mM Tris‐Cl pH 7.4, and 1 mM EDTA) and transferred to an Eppendorf tube. The suspension was centrifuged (5′, 3000 rpm), and the yeast pellet was resuspended in 250 µL of LiAc–TE solution.

In total, 50 µL of the resuspended cells were mixed with 280 µL of PEG 3500 (50% W/V), 70 µL of 5X LiAc–TE (0.5 M LiAc, 250 mM Tris‐Cl pH 7.4, 5 mM EDTA), and 25 µg of herring genomic ssDNA and 80 ng of plasmid.

In the modified protocol, 40 µL of 2 M sorbitol solution were added to this mix.

The tube was then incubated at 30°C for 30 min and heat‐shocked at 42°C for 15 min. After centrifugation (5′, 3000 rpm), yeast cells were resuspended either in 1 mL of water (standard protocol) or in 1 mL of 0.5 M sorbitol (modified protocol).

#### 4.2.1 Viability Assay

In total, 10 µL of this suspension were diluted in 1 mL of water or 2 M sorbitol, and this dilution was subsequently diluted 1:100 before plating 100 µL on YPD. After 3 days at 30°C, the number of colonies was counted. Statistical analyses were conducted using R.

#### 4.2.2 Transformation Efficiency

In total, 100 µL of this suspension were plated onto SC‐Trp. After 3 days at 30°C, the number of colonies was counted. Statistical analyses were performed using R.

## Author Contributions

Mathilde Kadouch, Pierre Gaspin, Christelle Marchal, Sabine Castano, and Christophe Cullin performed experiments and contributed to the review and editing of the manuscript. Mathilde Kadouch and Christophe Cullin wrote the paper. Christophe Cullin conceived the experiments.

## Conflicts of Interest

The authors declare no conflicts of interest.

## Data Availability

The data that support the findings of this study are openly available in Authorea at https://www.authorea.com/users/830990/articles/1224671-enhancing-yeast-transformation-achieving-a-10-fold-increase-through-a-single-adjustment-in-the-lithium-acetate-polyethylene-glycol-method?commit=488cb68b1692a59e1f9be793154b7c5d91eda2a0, reference number DOI: 10.22541/au.172618743.38549537/v1.
